# Trends in Developed Land Cover Adjacent to Habitat for Threatened Salmon in Puget Sound, Washington, U.S.A.

**DOI:** 10.1371/journal.pone.0124415

**Published:** 2015-04-29

**Authors:** Krista K. Bartz, Michael J. Ford, Timothy J. Beechie, Kurt L. Fresh, George R. Pess, Robert E. Kennedy, Melinda L. Rowse, Mindi Sheer

**Affiliations:** 1 Conservation Biology Division, Northwest Fisheries Science Center, National Marine Fisheries Service, National Oceanic and Atmospheric Administration, Seattle, Washington, United States of America; 2 Fish Ecology Division, Northwest Fisheries Science Center, National Marine Fisheries Service, National Oceanic and Atmospheric Administration, Seattle, Washington, United States of America; 3 College of Earth, Ocean, and Atmospheric Sciences; Oregon State University, Corvallis, Oregon, United States of America; Aberystwyth University, UNITED KINGDOM

## Abstract

For widely distributed species at risk, such as Pacific salmon (*Oncorhynchus* spp.), habitat monitoring is both essential and challenging. Only recently have widespread monitoring programs been implemented for salmon habitat in the Pacific Northwest. Remote sensing data, such as Landsat images, are therefore a useful way to evaluate trends prior to the advent of species-specific habitat monitoring programs. We used annual (1986-2008) land cover maps created from Landsat images via automated algorithms (LandTrendr) to evaluate trends in developed (50-100% impervious) land cover in areas adjacent to five types of habitat utilized by Chinook salmon (*O*. *tshawytscha*) in the Puget Sound region of Washington State, U.S.A. For the region as a whole, we found significant increases in developed land cover adjacent to each of the habitat types evaluated (nearshore, estuary, mainstem channel, tributary channel, and floodplain), but the increases were small (<1% total increase from 1986 to 2008). For each habitat type, the increasing trend changed during the time series. In nearshore, mainstem, and floodplain areas, the rate of increase in developed land cover slowed in the latter portion of the time series, while the opposite occurred in estuary and tributary areas. Watersheds that were already highly developed in 1986 tended to have higher rates of development than initially less developed watersheds. Overall, our results suggest that developed land cover in areas adjacent to Puget Sound salmon habitat has increased only slightly since 1986 and that the rate of change has slowed near some key habitat types, although this has occurred within the context of a degraded baseline condition.

## Introduction

Recovery of threatened species in their natural habitat typically involves identifying the factors responsible for the species’ at-risk status and developing and implementing plans to address those factors. For example, the U.S. Endangered Species Act of 1973 (7 U.S.C. § 136, 16 U.S.C. § 1531 et seq.) requires that federal agencies determine whether a species is threatened or endangered due to factors such as habitat loss, overutilization, inadequate regulation, or disease (ESA Section 4(a)(1)). In developing recovery plans for listed species, the ESA requires both a description of specific actions needed to address the factors that have led to its at risk status, and objective criteria that when met would result in the species no longer being at risk (ESA Section 4 (f)). The status of the species with respect to these criteria must be evaluated at least every five years (ESA Section 4(c)(2)), so trend monitoring of both the status of the species and the limiting factors is important.

Although species are threatened by a wide variety of human activities and natural factors, loss or degradation of habitat is a common cause of decline for many [1–4]. Therefore, habitat restoration is a common element of many recovery plans [5]. Monitoring of changes in habitat is also an objective of a majority of recovery plans, but implementation of such monitoring is often lacking [6]. For some species—particularly wide ranging species that occupy a variety of distinct habitats throughout their lives—monitoring changes in habitat can be a complex and expensive undertaking. ESA-listed Pacific salmon are a good example of a group of species for which habitat is clearly a factor limiting recovery, but for which monitoring changes in habitat quality is not straightforward.

Nine species of anadromous Pacific salmon and trout in the genus *Oncorhynchus* are native to the North Pacific Ocean, from Beringia to southern Japan in the west and southern California in the east. The ranges of seven of these species include streams, rivers, estuaries, and nearshore areas of the west coast of North America [7]. Although considerable life-history diversity exists both among and within these species, all have life cycles that involve reproduction in fresh water, migration of juveniles to the ocean for a period of feeding and growth, and a return migration of adults to natal streams to reproduce [8]. All Pacific salmon and trout species therefore rely to some extent on freshwater, estuarine, and nearshore marine habitats—areas that are especially vulnerable to pressures resulting from human occupation and development [9].

Monitoring the effects of some threats to salmon, such as harvest by fisheries or passage through dams, has become routine and institutionalized such that long term (decades or more) trends of changes in these factors are readily available [10,11]. By contrast, such decadal or longer term trends are rarely available for indices of freshwater or estuarine salmon habitat, although there are some exceptions (e.g., [12,13,14]). Reasons for the lack of routine habitat monitoring include the logistical difficulty of collecting data at many sites rather than at a few well-defined points, the need to coordinate collection protocols among multiple political jurisdictions, and the perceived high costs of collecting such data.

Developed land cover, consisting of roads, buildings, parking lots, and other impervious surfaces, has been associated with poor habitat conditions for salmon [15–18]. Trends in land cover may therefore be an important source of information for monitoring trends in salmon habitat [17]. Trends in land cover are also amenable to analysis through use of remote sensing data that are available at low or no cost to natural resource managers (reviewed by [19]). Focusing on land cover data may therefore be particularly useful for a retrospective analysis of trends in areas for which other data are unavailable or impractical to acquire for the time period of interest.

Here, we attempt to quantify a metric of long-term change in salmon habitat quality by analyzing trends in developed land cover in the areas adjacent to habitat utilized by ESA-listed Puget Sound Chinook salmon (*O*. *tshawytscha*). Although some previous studies have related land cover to salmon habitat in all or parts of Puget Sound (e.g., [17,20]), our study is unique in several respects. First, we focus on land cover trends during a time period—1986 to 2008—that encompasses the ESA listing in 1999, and ask whether there is any evidence for a change in trend during this time period. Second, we estimate separate land cover trends associated with five distinct types of salmon habitat (nearshore, estuary, mainstem river, tributary, and floodplain), which is important because different habitats may be subject to different levels of pressure from development. Third, we analyze land cover trends in the same way in each watershed, allowing comparisons of land cover change among watersheds. Finally, we utilize an analysis method that classifies land cover on an annual time step, a finer temporal scale than utilized previously.

### Study Site

**Fig 1:**
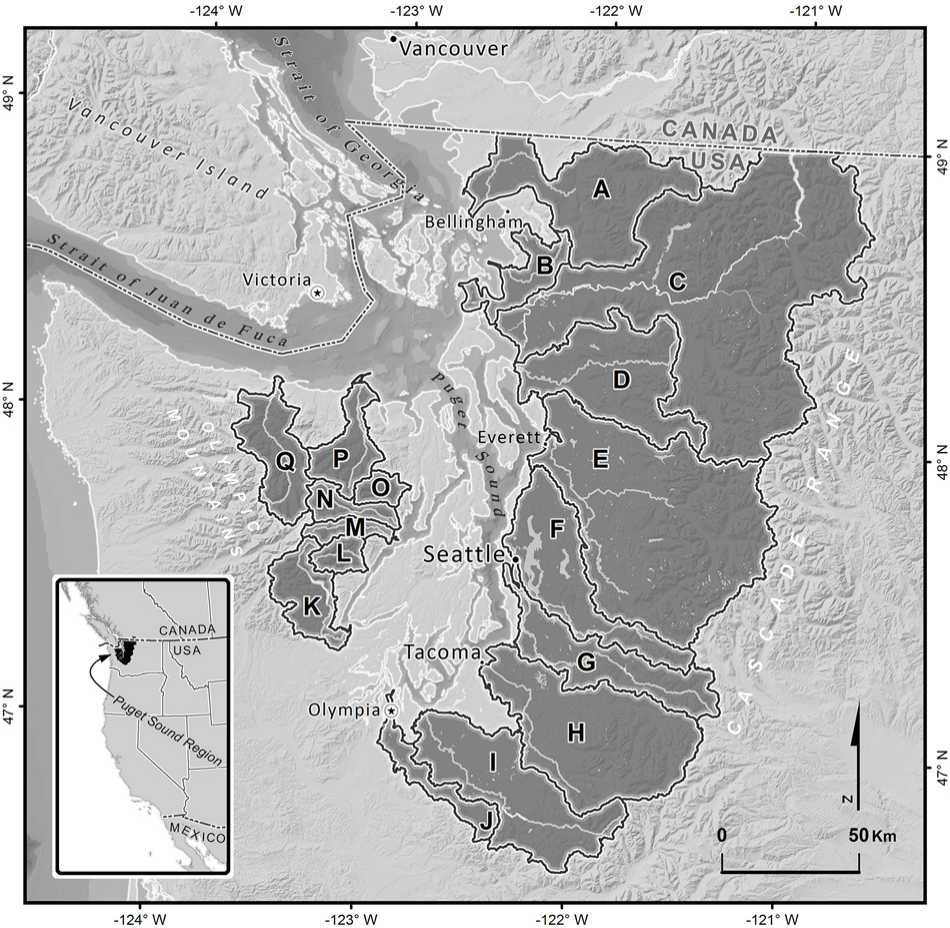
Puget Sound Region Map. Map of the Puget Sound region of Washington State (inset), including the 17 major watersheds analyzed in our study. A—Nooksack, B—Samish, C—Skagit, D—Stillaguamish, E—Snohomish, F—Lake Washington, G—Duwamish, H—Puyallup, I—Nisqually, J—Deschutes, K—Skokomish, L—Hamma Hamma, M—Duckabush, N—Dosewallips, O—Quilcene, P—Dungeness, Q—Elwha.

Puget Sound, located in the northwest corner of Washington State, U.S.A., is an inlet of the Pacific Ocean and part of the Salish Sea. It covers an area of approximately 6,400 km^2^ that is 160 km long and fringed by 3,700 km of coastline (Fig 1). The surrounding landscape includes 17 major watersheds that drain to the Sound from the Cascade and Olympic Mountain ranges. Collectively, these large watersheds and smaller ones between them comprise the Puget Sound region.

Human population size in the 9 counties of the Puget Sound region has grown from less than 300,000 in 1900 to more than 4,300,000 in 2010 [21,22]. Nearly 30% of that growth (more than 1,000,000 people) occurred during the period from 1990 to 2010, and population projections indicate that Washington State is expected to grow by >2,000,000 additional people by 2040 [23].

Puget Sound Chinook salmon were listed as threatened in 1999 and are one of 28 population groups of Pacific salmon currently listed under the ESA [24]. Loss and degradation of habitat in the Puget Sound region was a major factor contributing to the ESA listing [25]. In addition to Chinook salmon, populations of steelhead (*O*. *mykiss*) and chum salmon (*O*. *keta*) are also ESA-listed in the Puget Sound region [24]. As a result of these and other ESA listings of Pacific salmon, considerable resources have been devoted to habitat recovery. For example, from 2000 to 2011, the U.S. Congress funded an average of $79,000,000 annually to the Pacific Coast Salmon Recovery Fund, of which an average of $6,500,000 was spent annually on habitat recovery projects in Puget Sound [26]. The U.S. Environmental Protection Agency has also provided Washington State with over $30,000,000 since 2008 to aid in Puget Sound restoration, and state and local governments have devoted considerable staff and monetary resources toward salmon habitat restoration and monitoring. Despite these efforts, there are concerns that Puget Sound salmon habitat continues to be degraded and that restoration projects are not sufficient to offset continuing habitat loss [27,28]. Difficulties in evaluating these concerns are exacerbated by the lack of a comprehensive salmon habitat monitoring program in the Puget Sound region.

## Methods

Our study focused on two main questions: 1) what is the overall trend in developed land cover in the Puget Sound region during the two decades that span the ESA listing of Chinook salmon in the area? and 2) is there any evidence that the trend has changed during this time period? We answered these questions using a novel land cover dataset, focusing on locations adjacent to five salmon habitat areas: nearshore, estuary, mainstem, tributary, and floodplain. In short, we first delineated boundaries for the five habitat areas. We then quantified developed land cover by year and analyzed trends for each habitat area at two spatial scales: the Puget Sound region as a whole and within major Puget Sound watersheds.

### Land Cover

The land cover dataset for the Puget Sound region was derived from time-series analysis of Landsat Thematic Mapper and Enhanced Thematic Mapper + satellite images for the period 1986 to 2008. Landsat imagery has long been a standard tool for land cover mapping at regional scales, in large part because it balances relatively fine spatial resolution (30 x 30 m per pixel) with broad geographic coverage (180 x 180 km per image) and collects measurements in regions of the electromagnetic spectrum needed to distinguish among key land cover types [29]. Increasingly, the long, continuous record of observation (1980s onward) has been leveraged to extract rich historical information about the evolution of landscapes and landscape processes. This study builds on one such approach.

Yearly land cover maps were developed using outputs from a set of automated image processing algorithms called “LandTrendr” (Landsat-based Detection of Trends in Disturbance and Recovery) [30,31]. The LandTrendr algorithms examine how individual pixels change from year to year, distilling the time-series of pixel values into a progression of straightline segments that efficiently describe the entire temporal progression. While initially developed to identify segments associated with disturbance, the segmentation approach effectively removes much of the year-to-year noise caused by variation in vegetative seasonality, sun illumination angle, and residual atmospheric effects. These noise sources generally introduce false change in land cover maps. By removing such noise effects, the LandTrendr algorithm allows development of substantially more stable and consistent maps over time.

For this study, LandTrendr-stabilized images were used to develop yearly land cover maps based on training information derived from the 2001 National Land Cover Database (NLCD) [32] land cover maps. Because not all of the 16 original land cover classes in the NLCD maps were statistically separable, they were first aggregated into 7 classes that were distinguishable from spectral data alone: open water, snow/ice, barren land, deciduous forest, evergreen forest, herbaceous, and developed [33]. The LandTrendr “developed” class is associated most strongly with the medium-, and high-intensity developed NLCD classes, defined as 50–79%, and 80–100% impervious, respectively. Stabilized imagery from 2001 was linked with the aggregated NLCD land cover at a random sample of locations across the Puget Sound region, and the RandomForests classification algorithm [34] was used to model the relationship between spectral data and the land cover classes. Once established for 2001, this model was then applied to stabilized imagery from all other years between 1986 and 2008. At a random sample of locations across the region, interpreters validated land cover calls using available historical air photos; per-class accuracies for the developed and evergreen classes were approximately 64% and 69%, respectively [33]. Many classification errors involved misclassifications either within the four NLDC developed categories (which are combined in our analysis) or within the categories other than developed (also combined in our analysis). When only the categories of “developed” and “not developed” were considered, per-class accuracies were approximately 78% and 90%, respectively [33].

### Delineation of Habitat Analysis Areas

We identified areas of influence adjacent to five types of salmon habitat: nearshore, estuary, mainstem, tributary, and floodplain. For each type of habitat, we aligned definitions of the analysis area with those from other studies to the extent possible. Boundaries for each habitat area were defined as follows (see S1 File for additional details).

The nearshore analysis area extended 200 m inland from the ordinary high water mark of the marine shoreline [35,36]. It was bounded laterally by the seven Puget Sound sub-regions described in the same studies. The estuary analysis area included the 16 large river deltas that drain to Puget Sound. The boundaries encompassed historical wetland and intertidal areas, as well as areas draining directly to those wetlands or to the adjacent shoreline [35]. We defined mainstems as stream channels with bankfull widths >25 m to maintain consistency with other Puget Sound studies [37,38], and the analysis area was the riparian buffer extending 100 m landward from each channel bank [39,40], as identified in two hydrography datasets [41,42]. We defined tributaries as channels with bankfull widths of 5–25 m [37], and the tributary analysis area was also the riparian buffer extending 100 m landward from each bank. ESA-listed Chinook salmon primarily utilize large river systems in this region, so only tributaries draining to the 17 major river mainstems were included in the analysis (i.e., small channels draining directly to Puget Sound were omitted). The floodplain analysis area encompassed the area less than 10 m above the channel elevation in the 10-m National Elevation Dataset [43]. Only reaches with drainage areas >50 km^2^ were included; reaches with drainage areas below this threshold were considered too small to support significant amounts of floodplain habitat.

We also examined trends in each of the 17 major watersheds in their entirety (i.e., not broken down into the specific habitat areas describes above). This analysis area was delineated by aggregating the 5^th^ level hydrologic unit boundaries from the Watershed Boundary Dataset (http://water.usgs.gov/GIS/huc.html). Hereafter, it is called the “basin” analysis area to distinguish it from the “watershed” spatial scale in our analysis.

### GIS Methods

Boundaries for each habitat area were generated via geographic information systems (GIS) techniques, using ArcMap 10.1. Input layers and methods for generating the boundaries varied by habitat type (S1 File). All boundaries were re-projected to match the LandTrendr coordinate system. We then overlayed the LandTrendr data for a given year with the boundary of a given habitat area, and used the Tabulate Areas tool in the Spatial Analyst extension to quantify the area of developed land cover. Results were exported to Excel, and then summarized as hectares (ha) and percent (%) developed land cover.

### Statistical Methods

Statistical methods consisted of regression analyses of two types, consistent with the questions posed by this study. To answer the first question ― what is the overall trend? ― we fit simple linear regression models for each habitat area and spatial scale using the lm function in R:

$$y\text{ = }a_{0}\text{ + }b_{0}x$$ 1

in which *y* = hectares of developed land cover, *x* = year, and *a*_*0*_ and *b*_*0*_ were the intercept and slope, respectively. We then examined the sign and significance of the slope coefficient (*b*_*0*_) and calculated the Akaike Information Criterion corrected for small sample size (AICc) [44]. In order to compare trends among watersheds and habitat areas of varying sizes, we also used percent developed land cover as the dependent variable (*y*).

To answer the second question ― has the trend changed? ― we fit segmented linear regression models (e.g., [45]) using the Segmented package in R [46]. In segmented models, the relationship between the dependent and independent variables is estimated by two or more straight lines connected at estimated “breakpoints.” In the case of a single breakpoint, the Segmented package uses an iterative method to estimate the parameters in Eq 2 below:

$$y\text{ = }a_{1}\text{ + }b_{1}x\;for\;x<\psi \text{ , }y\text{ = }a_{2}\text{ + }b_{2}x\;for\;x\text{ > }\psi$$ 2

where *y* and *x* are the dependent and independent variables, respectively; *a*_*1*_ and *b*_*1*_ are the left segment intercept and slope, *a*_*2*_ and *b*_*2*_ are the right segment intercept and slope; and *ψ* is the breakpoint where the two lines connect. Note that because the lines intersect at the breakpoint, there are only four free parameters in the two-segment model and the fifth (*a*_*2*_, in our case) can be calculated from the other four. We fit a segmented model with a single breakpoint for each combination of habitat type and scale, and then calculated the AIC*c*. Segmented regression models with AICc values at least 4 units lower than the AICc values for the corresponding simple regression models (i.e., Δ AICc >4), were considered to provide a substantially better fit to the data. Models with breakpoints in the tail ends of the 20-year time series (i.e., 1986–1990 or 2004–2008) were viewed with caution because of the difficulty in fitting a line to so few data points.

## Results

### Puget Sound Scale

#### Overall Trends

**Fig 2:**
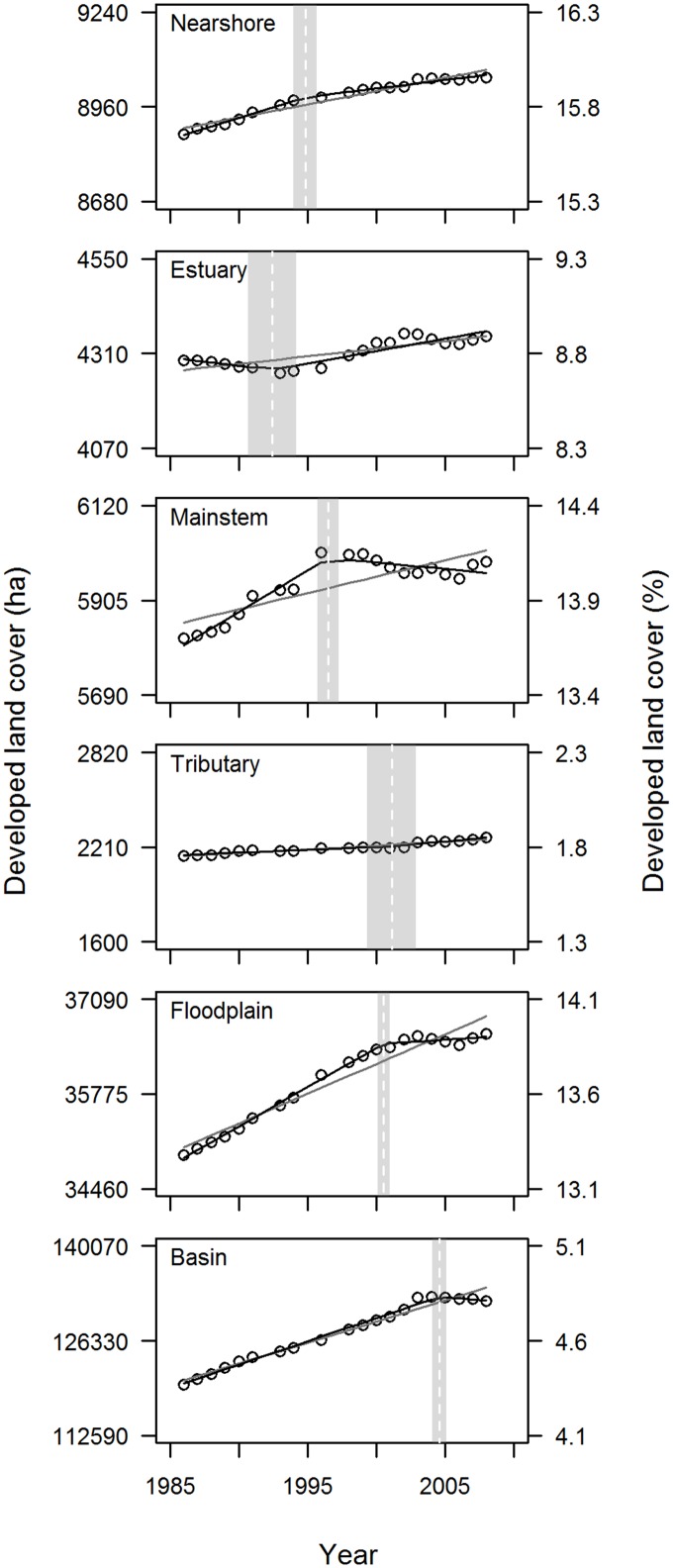
Trends in Developed Land Cover: Puget Sound Scale. Change over time in developed land cover, summarized at the Puget Sound scale for six habitat areas. Developed cover is depicted in ha (left axis) and % (right axis), and two types of regression models are fit to the data: simple (gray line) and segmented (black line). For the segmented model, the breakpoint estimate and standard error are also shown (dotted white line and gray rectangle, respectively).

According to our analysis, developed land cover increased from 1986 to 2008 for all habitat areas (Table 1, Fig 2). Slopes of the simple regression models were consistently positive (p <0.001; Table 2) but relatively small (<1 percentage point total increase from the beginning to the end of the time series; Table 1).

**Table 1:** Developed Land Cover in 1986 and 2008: Puget Sound Scale.

	Developed cover (ha)		% Change ^a^	Developed cover (%)		Change in % ^b^
Location	1986	2008		1986	2008	
Nearshore	8,876.97	9,047.25	1.9	15.66	15.96	0.30
Estuary	4,293.18	4,354.83	1.4	8.81	8.94	0.13
Mainstem	5,819.67	5,991.66	3.0	13.64	14.04	0.40
Tributary	2,153.34	2,272.05	5.5	1.78	1.88	0.10
Floodplain	34,935.48	36,609.93	4.8	13.32	13.96	0.64
Basin	119,969.01	132,135.30	10.1	4.37	4.81	0.44

Developed land cover (as hectares or percent of total area) in 1986 and 2008, summarized at the Puget Sound scale for six habitat areas.

^a^ Calculated as the difference in developed cover (in ha) between 2008 and 1986, divided by the developed cover in 1986, and expressed as a percentage.

^b^ Calculated as the difference in developed cover (%) between 1986 and 2008.

**Table 2:** Simple Regression Analysis Results: Puget Sound Scale.

	Intercept (*a*_*0*_)		Slope (*b*_*0*_)			
Location	Coefficient	SE	Coefficient ^a^	SE	*R* ^2^	*p*
Nearshore	-6,820.99	841.32	7.91	0.42	0.95	<0.01
Estuary	-3,533.41	1,298.80	3.93	0.65	0.67	<0.01
Mainstem	-8,756.43	2,490.79	7.36	1.25	0.66	<0.01
Tributary	-7,095.47	651.68	4.66	0.33	0.92	<0.01
Floodplain	-128,513.88	9,910.55	82.35	4.96	0.94	<0.01
Basin	-1,093,483.26	47,591.04	611.31	23.83	0.97	<0.01

Model coefficients (*a*_*0*_ and *b*_*0*_), standard errors (SE), and *R* ^2^ and *p*-values from fitting simple regression models to time series of developed land cover in six habitat areas, at the Puget Sound scale.

^a^ Slope units are hectares of developed land cover per year.

#### Changes in Trend

The segmented regression models consistently outperformed the simple regression models, according to their Δ AIC*c* values (Table 3), indicating changes in trend between 1986 and 2008 in all habitat areas. These shifts in trend followed three general patterns (Fig 2, Table 4). In estuary and tributary areas, the rate of increase in developed land cover accelerated after the breakpoint (i.e., *b*_*2*_ > *b*_*1*_). In nearshore and floodplain areas, the rate of increase slowed after the breakpoint (i.e., *b*_*2*_ < *b*_*1*_) but remained positive. Finally, in mainstem areas as well as whole basins, the rate of increase in developed land cover became flat or slightly negative after the breakpoint.

**Table 3:** Akaike’s Information Criterion for Simple and Segmented Regression Analyses: Puget Sound Scale.

Location	Simple AICc	Segmented AICc	Δ AICc ^a^
Nearshore	165.33	139.49	25.84
Estuary	182.70	177.09	5.61
Mainstem	208.74	178.99	29.75
Tributary	155.11	150.74	4.37
Floodplain	263.98	218.98	45.01
Basin	326.75	305.90	20.84

Akaike’s Information Criterion corrected for small sample sizes (AICc), calculated for two regression models (simple and segmented) in six habitat areas, at the Puget Sound scale. The difference in AICc (where Δ AICc = simple AICc—segmented AICc) is also provided.

^a^ Δ AICc >4 indicates support for the segmented regression model.

**Table 4:** Segmented Regression Analysis Results: Puget Sound Scale.

	Intercept (*a*_*1*_)		Slope (*b*_*1*_) ^a^		Slope (*b*_*2*_) ^b^			
Location	Coeff.	SE	Coeff.	SE	Coeff.	SE	Breakpoint	*R* ^2^
Nearshore	-16,278.22	1,652.77	12.67	0.83	5.10	0.50	1994.84	0.99
Estuary	12,763.00	7,531.32	-4.26	3.79	6.40	0.93	1992.45	0.82
Mainstem	-31,156.39	3,481.40	18.61	1.75	-2.91	1.58	1996.52	0.95
Tributary	-4,942.46	910.34	3.58	0.46	8.06	1.55	2001.11	0.95
Floodplain	-180,837.25	5,612.38	108.62	2.82	12.82	6.97	2000.53	1.00
Basin	-1,219,449.59	33,533.37	674.53	16.81	-153.25	177.40	2004.59	0.99

Model coefficients (a_*1*_, *b*_*1*_, and *b*_*2*_), standard errors (SE), breakpoints, and *R* ^2^ values resulting from fitting segmented regression models to time series of developed land cover in six habitat areas, at the Puget Sound scale.

^a^ *b*_*1*_ represents the slope for the first part of the time series (1986-breakpoint year) in ha/year.

^b^ *b*_*2*_ represents the slope for the second part of the time series (breakpoint year-2008) in ha/year.

### Watershed Scale

#### Overall Trends

**Fig 3:**
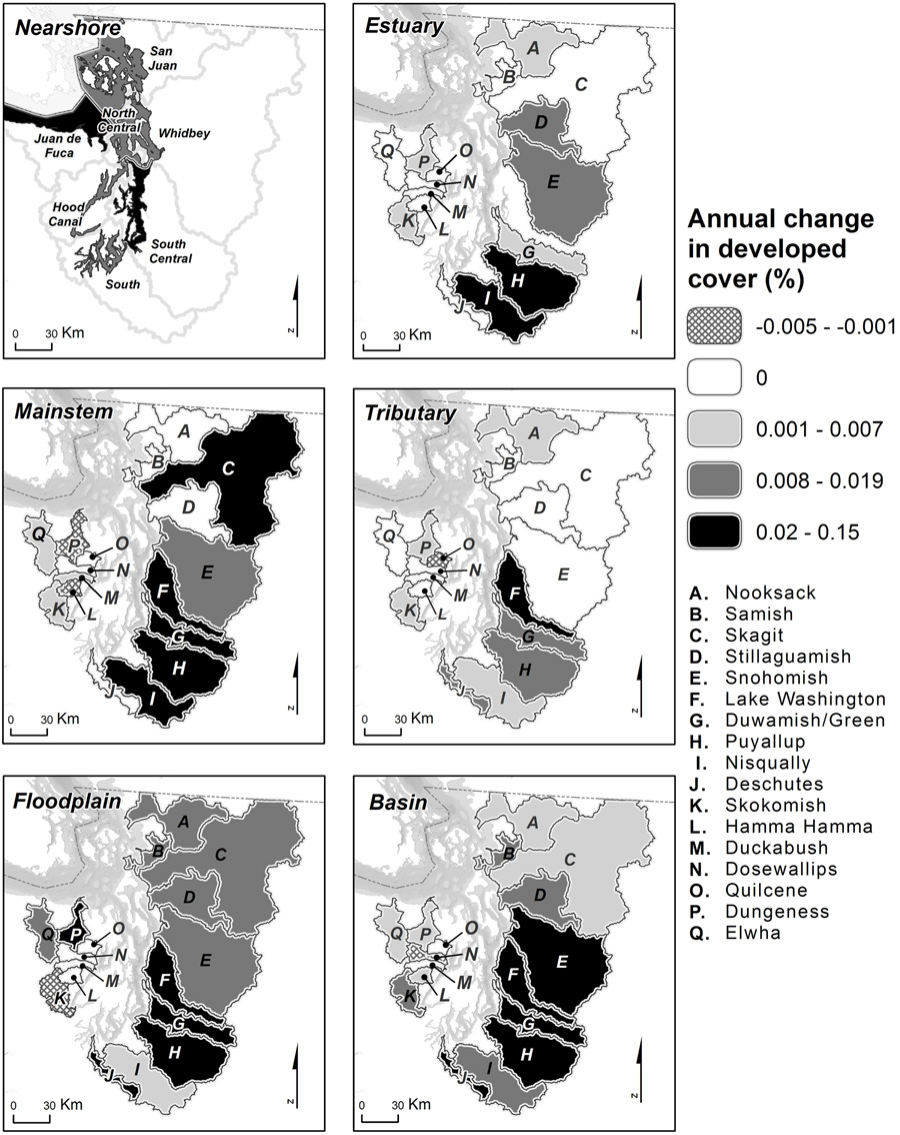
Annual Change in Developed Land Cover (*b*_*0*_): Watershed Scale. Annual rate of change in the percentage of developed land cover between 1986 and 2008 (*b*_*0*_ in Eq 1), summarized at the watershed scale for six habitat areas. Maps are shaded to depict the rate of change for a habitat area within a given watershed, but in each case the entire watershed is shaded to better visualize differences among watersheds. For the nearshore panel, the entire marine sub-region associated with each nearshore habitat area is shaded.

The Puget Sound-wide trends obscured considerable variation among individual watersheds. Developed land cover increased at least slightly from 1986 to 2008 in most basins, with the exception of several smaller basins on the west side of Puget Sound (Fig 3, S2 File). For areas surrounding specific types of habitat, however, trends were much more variable. Developed land cover increased least in areas surrounding tributary habitat, with regression slopes (*b*_*0*_) in northern and western watersheds either remaining flat or increasing <0.007% per year. However, slopes in southern watersheds increased more steeply. Developed land cover in nearshore areas increased at a similar rate for all watersheds. In contrast, trends in estuary areas were highly variable, with some watersheds having flat slopes (e.g., Skagit, Elwha) and others increasing slopes (e.g., Puyallup, Nisqually). Developed land cover in mainstem and floodplain areas increased for most watersheds, with the steepest slopes (≥0.020%) generally occurring in southern watersheds. As with the Puget Sound scale results, these changes occurred within a relatively narrow range (<2 percentage point increase or decrease in developed land cover during the course of the time series; S2 File).

**Fig 4:**
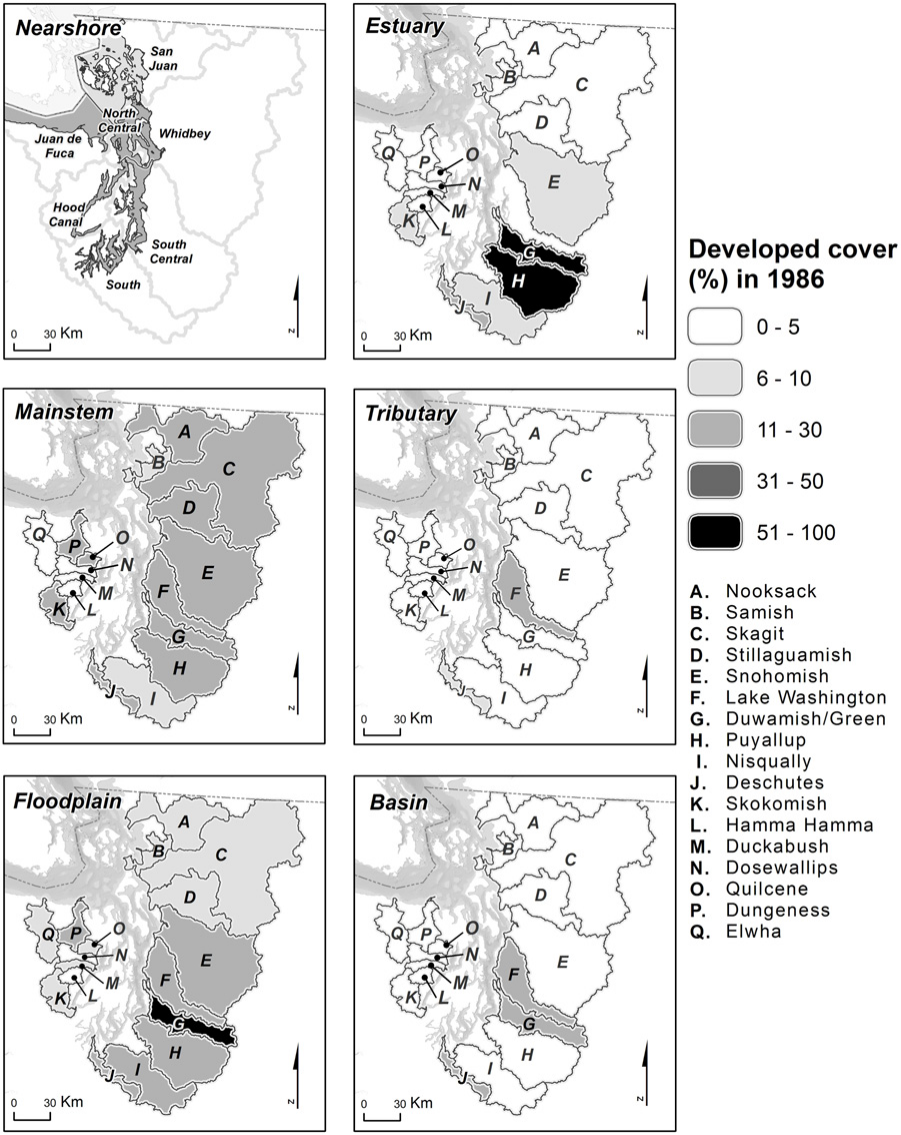
Percentage of Developed Land Cover in 1986: Watershed Scale. Percentage of developed land cover at the start of the time series in 1986, summarized at the watershed scale for six habitat areas. Format is identical to that in Fig 3.

**Fig 5:**
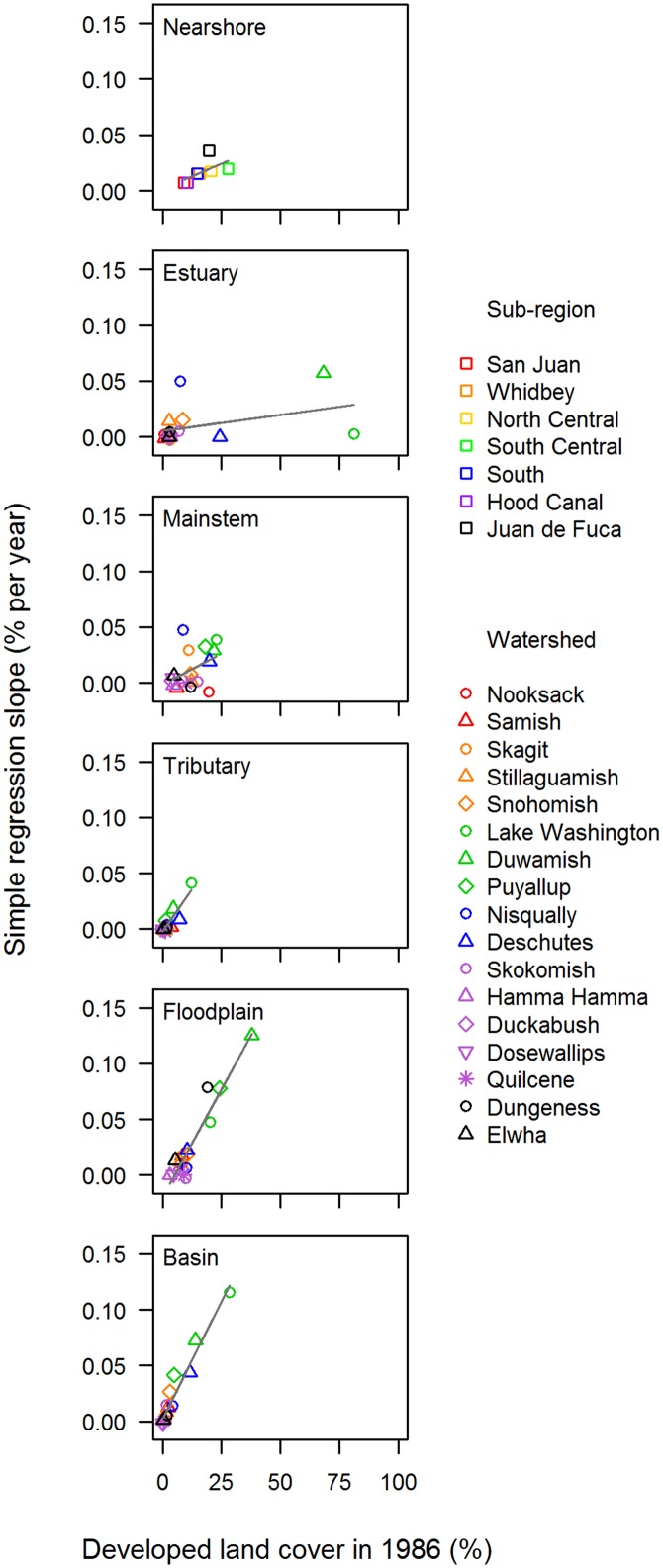
Percentage of Developed Land Cover in 1986 vs. Annual Change in Developed Land Cover (*b*_*0*_): Watershed Scale. The relationship between the percentage of developed land cover in 1986 and the annual rate of change in the percentage of developed cover (*b*_*0*_ in Eq 1), for each of six habitat areas. The line in each panel illustrates the fitted least-squares linear relationship.

In evaluating trends at the watershed scale, it should be noted that each watershed had a different baseline level of developed land cover in 1986 (Fig 4). These differences were particularly pronounced in estuary habitat areas. For example, some watersheds had >50% developed land cover in estuary areas in 1986, while others had <5%. In contrast, nearly all watersheds started the time series with 11–30% developed land cover in nearshore and mainstem habitat areas, and relatively little (<5%) in tributary habitat areas. For three of the six habitat areas ― tributaries, floodplains, and basins as a whole ― there was a strong positive relationship between the simple regression slope (*b*_*0*_) and the percentage of developed land cover at the start of the time series in 1986 (Fig 5; *R* ^2^ = 0.81, 0.90, and 0.94, respectively; all *p*-values < 0.01).

#### Changes in Trend

**Fig 6:**
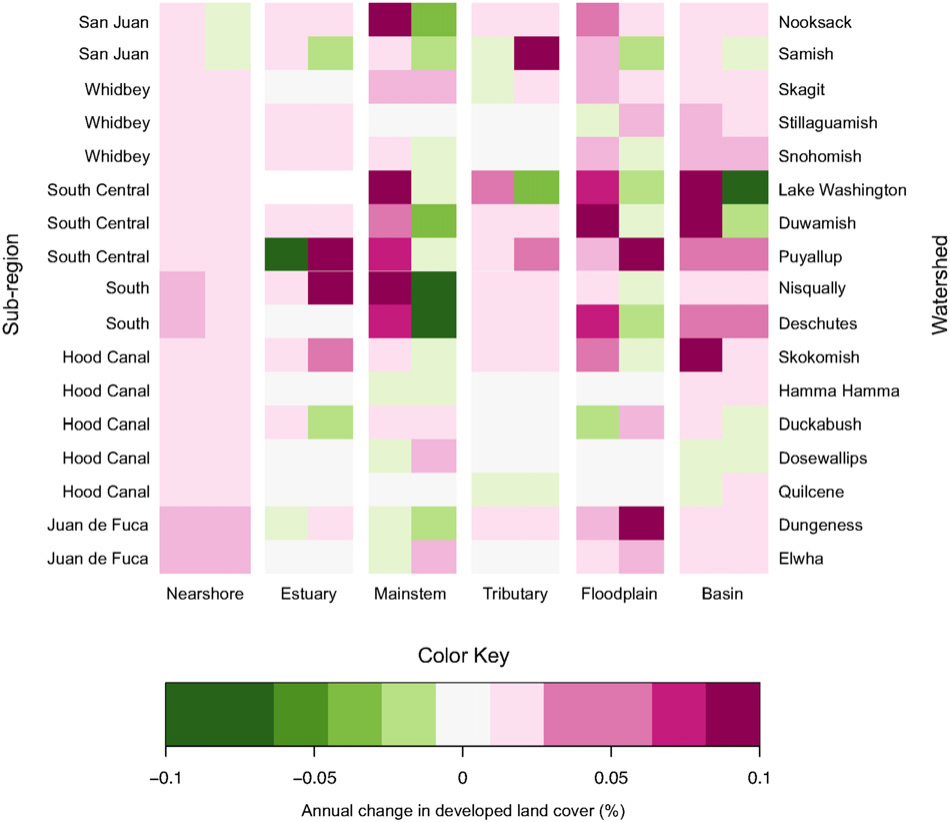
Changing Trends in Developed Land Cover: Watershed Scale. Heat map illustrating trends in developed land cover (regression slopes), summarized at the watershed scale for six habitat areas. Warmer colors indicate increasingly positive slopes; cooler colors increasingly negative slopes. A change in cell color between the first and second cell within a habitat-by-watershed combination indicates a substantially better fit of the segmented regression, compared to the simple regression (i.e., Δ AICc >4), hence *b*_*1*_ and *b*_*2*_ are shown. If Δ AICc was <4, both cells depict *b*_*0*_ for the simple regression, unless the *b*_*0*_ *p*-value exceeded 0.05, in which case both cells were assigned *b*_*0*_ = 0. Note that the North Central sub-region has no corresponding watershed and, therefore, was omitted (but see S2 File).

**Fig 7:**
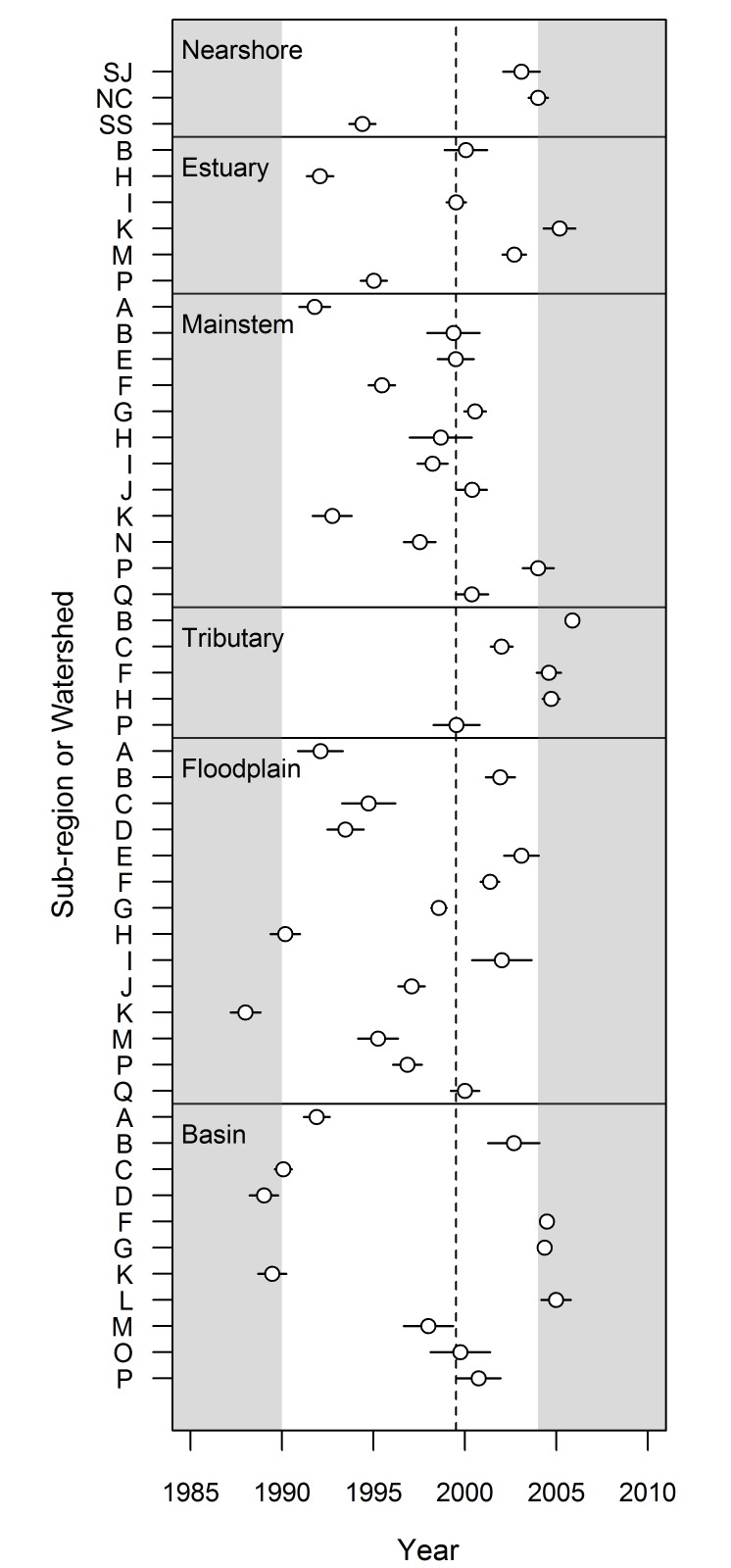
Breakpoint Estimates: Watershed Scale. Breakpoint estimates (open circles), standard errors (surrounding bars), and median value (dotted line) for six habitat areas, summarized at the watershed scale. Only those watersheds in which the segmented regression outperformed the simple regression (i.e., Δ AICc >4) are shown. Breakpoints within the tail ends of the time series (gray shaded areas) should be viewed with caution.

**Fig 8:**
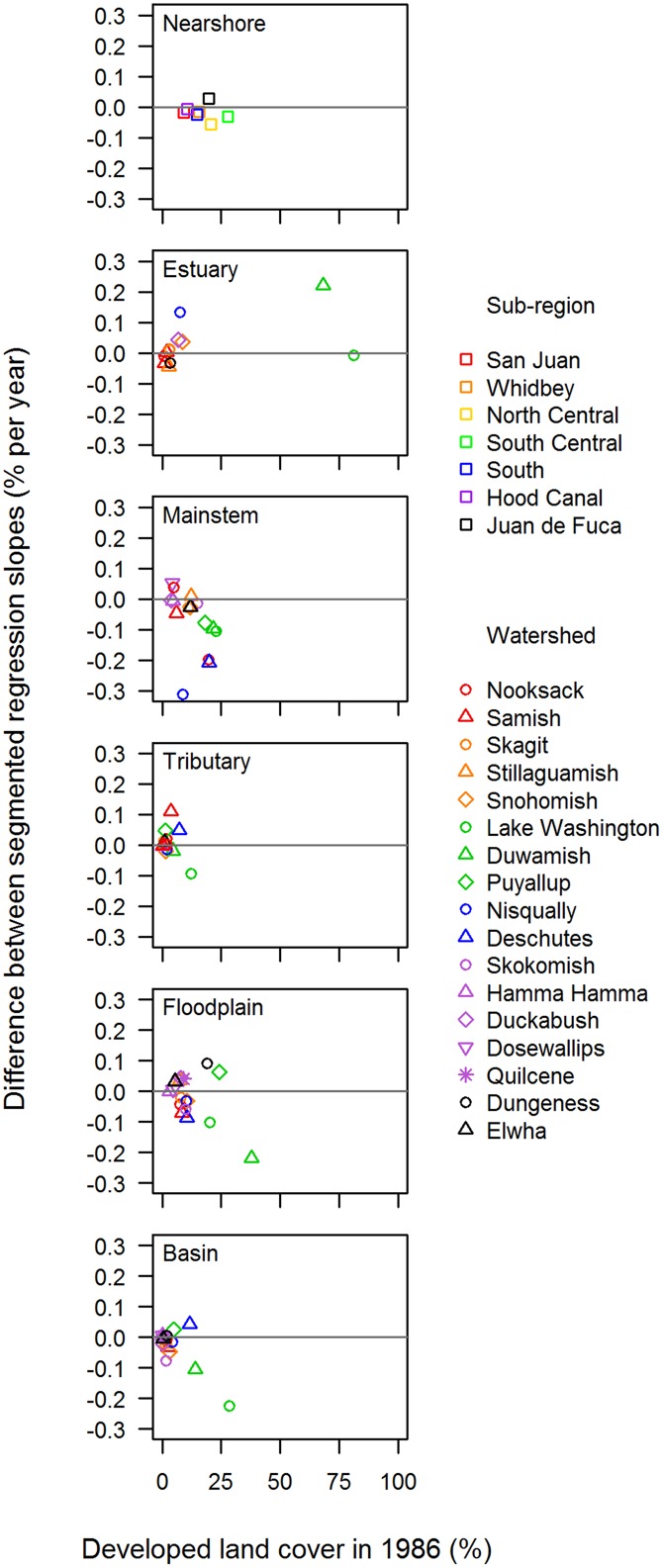
Percentage of Developed Cover in 1986 vs. Annual Change in Developed Land Cover (*b*_*2*_ *– b*_*1*_): Watershed Scale. The relationship between the percentage of developed land cover in 1986 and the difference in the annual rate of change in the percentage of developed cover before and after the breakpoint (*b*_*2*_ *– b*_*1*_ in Eq 2), for each of six habitat areas. Positive values on the *y*-axis indicate that the slope of the right-hand segment (latter part of the time series) exceeds the slope of the left-hand segment (earlier part of the time series); negative values indicate the opposite.

In total, we assessed changes in trend for 91 habitat-by-watershed combinations (i.e., 7 nearshore, 16 estuary, and 17 in each of the mainstem, tributary, floodplain, and basin areas). For 51 of these 91 combinations, Δ AIC*c* values indicated strong evidence for breakpoints in trends at the watershed scale (Fig 6, S2 File). The timing of the breakpoint in trends varied greatly among watersheds and habitat areas (median = 1999.5), with no apparent pattern (Fig 7). In general, changes in trend were most dramatic in the central and southern watersheds. Trends in developed land cover adjacent to mainstem and floodplain habitats became flatter in the latter part of the time series for most watersheds (i.e., *b*_*2*_ < *b*_*1*_). Particularly for mainstem habitat, several watersheds (Nooksack, Nisqually, Deschutes) had substantial changes in trend, shifting from strongly increasing to strongly decreasing (Fig 6). The relationship between the degree of change in trend and the percentage of developed land cover in 1986 was complex, and varied considerably among habitat areas (Fig 8).

## Discussion

Our study examined the question of whether salmon habitat degradation has continued in the Puget Sound region during recent decades, despite the considerable resources and public awareness spurred by sharp salmon declines in the 1980’s and multiple salmon ESA listings in the 1990’s. Answering this question is not straightforward, in large part due to the absence of a coordinated salmon habitat monitoring program during this time frame. The indicator presented here ― developed land cover as measured through Landsat data ― is associated with degradation of salmon habitat [15,17], and is therefore a useful correlative metric to address this question. Focusing the analysis on five distinct types of salmon habitat captures spatially explicit trends and enables the development of hypotheses relating land cover change in each habitat type to Chinook salmon productivity. Our results will be of relevance to other salmon species in areas where their distributions overlap with Chinook salmon.

Despite an increase in human population size in the Puget Sound region of more than 1,000,000 people from 1990 to 2010 (>30% increase), developed land cover in all habitat areas increased by considerably less than 1 percentage point during approximately the same time frame (Table 1). Unlike previous studies that relied on only 2–3 time points (e.g., 2001 and 2006 in [27]), our study used 20 years of annual data. We could therefore directly assess whether trends have changed over time. This assessment produced one of our most notable results: for nearshore, mainstem, and floodplain habitat areas, and for basins as a whole, there was clear evidence of a flatter trend in developed land cover in the later part of the time series than in the earlier, at the regional scale (Fig 2). If formerly increasing trends in developed land cover in these areas have slowed or even in some cases reversed, that would be an important consideration for the conservation status of threatened salmon in Puget Sound. In particular, preventing further degradation of existing habitat was identified as an important but largely unevaluated goal in the Puget Sound Chinook Salmon Recovery Plan [47]. Our analysis focuses on only one indicator of habitat degradation, but, to the degree that trends in this indicator have improved over time, it is encouraging as it suggests that habitat protection efforts may be effective.

Factors that might cause formerly increasing trends in developed land cover to flatten include changes in human population growth, federal ESA listings, state or local regulations, overall economic climate, or increase of vegetative cover within existing developed areas. Each of these is a plausible contributor in limiting the rate of conversion to developed land cover, although none are likely to be the sole driver. For example, human population growth in the Puget Sound region was greater between 1990 and 2000 than between 2000 and 2010 [22], but the difference was not dramatic (a 20% increase versus a 13% increase, respectively). Restrictions on development or increased regulatory uncertainty associated with the ESA listing of Puget Sound Chinook salmon in 1999 might have slowed development, but many of the breakpoints preceded that year (Figs 2 and 7). Similarly, the passage of Washington State’s Growth Management Act in 1990 might have contributed to flattening rates of development, but its local implementation on varying schedules would have extended its influence throughout our study period. Washington State also experienced a flattening in the trend of household incomes from 1998 to 2002 [48] which might be expected to slow development, but incomes rebounded from 2002–2008.

Additionally, although conversion to developed land cover is often perceived from the ground as a permanent change, from space the spectral signature of developed cover can change over time due to growth of vegetation. For example, a new housing development will have more pixels classified as developed than the same development after trees and shrubs have matured. It is therefore possible that increased planting or vegetative growth within developed areas could contribute to the observed trends. This seems particularly likely in habitat areas where developed land cover declined slightly over time. Vegetation within urban areas may be relatively ineffective at ameliorating the effects of widespread impervious surfaces [18], primarily because ecological impacts are significant when impervious surface cover reaches 10% and most urban areas are well above that 10% threshold. Therefore, if flattening trends are largely due to vegetative growth within already urbanized areas, these trends may not be indicative of improving habitat quality for salmon.

A clear, positive relationship existed at the watershed scale between the rate of development *(b*_*0*_) and the degree to which tributary, floodplain, and basin areas were already developed in 1986 (Fig 5). This pattern suggests that, at least from 1986 to 2008, development was concentrated in areas that likely already had degraded salmon habitat. Concentrating development in already developed areas is a goal of the Washington State Growth Management Act (Wash. Rev. Code § 36.7A), and also is broadly consistent with the Puget Sound Chinook salmon recovery strategy [47].

Increasing trends in developed cover during the early part of the time series tended to be steepest in watersheds that were already highly developed, especially in mainstem and floodplain areas of central and southern Puget Sound. However, many of these highly developed areas actually had flat or decreasing trends in developed land cover during the latter part of the time series (Figs 6 and 8), suggesting either a potential shift in land management near large rivers and in floodplains or perhaps a saturation of development. For example, mainstem and floodplain areas of the Lake Washington, Duwamish, Puyallup, and Deschutes watersheds all had relatively large increases in developed land cover during the early part of the time series (Fig 6), and these habitat areas already contained relatively high proportions of developed cover at the start of the time series (Figs 4 and 8). These same areas, with the exception of the Puyallup floodplain, had decreasing trends in developed land cover during the latter part of the time series (Fig 6).

Floodplain and mainstem development is associated with loss of floodplain habitat and armoring of stream banks [49,50], both of which reduce habitat capacity for juvenile salmon [49,51]. Hence, habitat capacity has been significantly reduced in mainstem and floodplain areas with high percentages of developed land cover. Recent declines in developed land cover may therefore signal modest improvements in habitat capacity if those declines are a result of floodplain or mainstem restoration actions, but these improvements may be limited because the baseline level of development remains high.

Our results do not indicate that Puget Sound salmon habitat is currently in good condition. We have shown, however, that the changes in developed land cover since 1986 have been relatively small, and what had been an upward trend in developed land cover appears to have been ameliorated for some habitat areas. Developed land cover in many habitat areas exceeds 10% (Table 1, Fig 4, S2 File), which might indicate permanent degradation of salmon habitat [18]. In addition, despite flattening or even improving trends for some key habitat areas (Figs 2 and 5), the overall trend for most habitat areas was a net increase in developed cover over the course of the time series and this change has occurred in the context of a degraded baseline condition (Table 1, Fig 4). Particularly in estuary, mainstem, and floodplain habitat areas, most watersheds had already experienced substantial conversions to commercial, residential, agricultural, or other human uses during the hundred years prior to the start of our time series in 1986 [52]. The relatively small changes in developed land cover we detected in the subsequent 22 years are, therefore, in addition to much larger changes that occurred earlier.

It is also important to view these trends in the context of accuracy of the land cover mapping algorithms. Overall accuracies of the two main classes of interest here (developed and not developed) were reasonable (78% and 90%) [33], but these accuracies would not be high enough to accurately detect subtle changes in cover in a simple comparison of independently classified land cover maps. However, in our study land cover class will only change if the core LandTrendr algorithm detects a change in spectral signal, regardless of its land cover classification status [30]. Therefore, the accuracy of change in land cover is expected to be much greater than the accuracy of any single annual static land cover classification. The fact that the annual variance in classification is small (Fig 2) suggests this expectation is being met for our data set. Thus, we believe that overall trends in the developed land cover class are reasonable approximations of what has actually occurred.

Our results differ somewhat from estimates obtained from broader scale, sample-based, land cover change analyses that included the Puget Sound region. For example, in a national scale analysis of land cover change [53], it was estimated that developed cover in the marine west coast forest region as a whole increased by ~1.25 percentage points between 1986 and 2000, about three-fold more than our estimated increase of 0.44 percentage point for the Puget Sound basin over a longer time period (Table 1). Similarly, in a national-scale analysis of riparian land cover change [54], a relatively large loss of natural riparian cover (~4 percentage points) was estimated for the Puget Sound region from the mid-1980’s to the early 1990’s, although the rate of loss was greatly reduced after this time period (like our results for mainstem habitat). Our study focused on both the entire and select subsets of the Puget Sound region and was based on higher resolution (individual pixel trend) information than these broad surveys, so our estimates for developed land cover are likely to be more accurate for the specific habitat areas we studied.

The human population of the Puget Sound region has grown markedly during the past several decades, but the degree of increase in developed land cover in the region since 1986 appears to be considerably less than what has been observed in some other fast growing parts of the United States. For example, a similar pixel-based methodology as ours was used to examine trends in impervious land cover in the Washington, D.C./Baltimore, MD region over a similar time period (1984–2010), and it was found that impervious cover increased 1.2 percentage points (3.7% to 4.9%) with no obvious change in the trend over that time [55]. The coastal Washington/Baltimore region analyzed is of similar size to the Puget Sound region and is similarly characterized by a large inlet (Chesapeake Bay), but the trend of development appears to be somewhat different. The Seattle and Washington D.C. metropolitan areas are both characterized by relatively slow rates of urban land cover growth compared to other international cities [56], and the differences between the two areas are likely to be related primarily to differences in growth outside of the urban cores.

Quantifying trends in developed land cover adjacent to marine, estuarine, and fresh waters is an important first step toward developing a comprehensive monitoring program for habitats associated with ESA-listed salmon species. As an indicator of salmon habitat quality, developed land cover has certain limitations that need to be kept in mind when interpreting our results. In particular, other types of land use, such as agriculture and forestry, are associated with salmon habitat degradation [17], but are classified as “herbaceous” or “barren” rather than “developed” by LandTrendr, and are therefore not captured in our analysis. In addition, much of the funding for salmon habitat restoration has been applied toward improvements in areas not considered as “developed” in our analysis [57]. Placement of log structures in mainstems, reconnection of side channels in floodplains, and conversion of agricultural lands to wetlands are examples of the types of habitat improvements that are not reflected in our analysis. Clearly, therefore, a complete evaluation of trends in salmon habitat should include other indicators that will be sensitive to changes not captured by changes in developed land cover. This study provides a useful starting point, however, for evaluating the changes in land cover adjacent to salmon habitat in Puget Sound and will provide a valuable template for similar analyses in other areas with threatened salmonids.

## Supporting Information

S1 File Methods for Developing Habitat Analysis Area Boundaries. (DOCX)

S2 File Watershed Scale Results, Comparable to Tables 1–4. (XLSX)
