# Supplementary material for: Trends in Developed Land Cover Adjacent to Habitat for Threatened Salmon in Puget Sound, Washington, U.S.A
Source: PLoS One. 2015 Apr 29;10(4):e0124415. doi: 10.1371/journal.pone.0124415 (PMC4414273; doi:10.1371/journal.pone.0124415)
Supplement: S1 File — (DOCX) [file pone.0124415.s001.docx]

**Methods for Developing Habitat Analysis Area Boundaries**

Nearshore Habitat Area

Boundaries for the nearshore habitat area matched those developed by the Nearshore Science Team of the Puget Sound Nearshore Ecosystem Restoration Project (PSNERP). The boundaries are part of a broader geodatabase [1,2] available at http://wagda.lib.washington.edu/data/geography/wa_state/#PSNERP. Our workflow in ArcMap involved:

1. selecting polygons with the following attributes from the fd_GSU layer:
   1. ZU = 1
   2. DPU_1 and DPU_2 = null
   3. DPU2 = STL, if bordering Camano Island; and
2. exporting those 4751 polygons to create a new data layer.

This new data layer contained only nearshore polygons, defined as the area extending 200 m inland from the ordinary high water mark of the marine shoreline. We used the SUBBASIN attribute in this layer to aggregate results at the scale of 7 marine sub-regions (San Juan Islands/Georgia Strait (SJ), Whidbey Basin (WH), North Central Puget Sound (NC), South Central Puget Sound (SC), South Puget Sound (SP), Hood Canal (HC), and Strait of Juan de Fuca (JF)). The 200 polygons with a split SUBBASIN designation (e.g., HC|NC) were assessed visually and then assigned either to the first sub-region (in the case of 191 polygons) or the second sub-region (in the case of 9 polygons) listed.

Estuary Habitat Area

Estuary boundaries were derived from the same PSNERP geodatabase as the nearshore boundaries. The workflow, therefore, was similar to that described above in that it involved:

1. selecting polygons with the following attributes from the fd_GSU layer:
   1. ZU = 2
   2. DPU_1 and DPU_2 = not null; and
2. exporting those polygons to create a new data layer.

The new data layer contained only the 16 large river deltas that drain to Puget Sound. We used the DPU_1 and/or DPU_2 attributes in this layer to aggregate results by estuary. Polygons for which DPU1 = SKG (Skagit) and DPU2 = STL (Stillaguamish) were included in the Skagit boundary.

Mainstem Habitat Area

To maintain consistency with other Puget Sound studies, mainstems were defined as stream channels with bankfull widths (bfw) >25 m. Mainstems were derived from two stream datasets: the Washington Waterbody Dataset (WBD; https://fortress.wa.gov/dnr/app1/dataweb/dmmatrix.html#Hydrography) and the high resolution National Hydrography Dataset (NHD; http://nhd.usgs.gov/data.html). The WBD ─ a polygon layer ─ was used wherever it was available. However, its extent was limited in many watersheds, so the NHD ─ a line layer ─ was used as a supplement. Neither layer contained bfw information. Therefore, a Puget Sound wide derived stream network (DSN) attributed with modeled bfw [58, cited below] was employed to identify channels with bfw >25 m in ArcMap by:

1. selecting (“by attributes”) DSN reaches with bfw >25 m;
2. selecting (“by location”) WBD or NHD reaches within 150 m of the selected DSN reaches;
3. adding a field in the WBD and NHD layers to identify which reaches were selected; and
4. editing this field to remove any mistakenly selected reaches (e.g., tributary reaches <25 m bfw located within 150 m of mainstem reaches).

To the mainstem reaches identified above, we then added a riparian buffer extending 100 m landward from each bank via the ArcMap “Buffer” tool. Next, we merged the buffered WBD and NHD layers via the “Union” tool, and performed a series of final steps to identify and omit extraneous polygons. For example, the “Identity” tool was used to distinguish mainstem areas that overlapped nearshore and estuary areas, as defined above. These areas of overlap were not included in subsequent analyses.

Tributary Habitat Area

Tributaries were defined as channels 5 to 25 m in bfw [3] and were derived from the Washington Watercourse Dataset (WCD; https://fortress.wa.gov/dnr/app1/dataweb/dmmatrix.html#Hydrography). The WCD, like the WBD and NHD described above, contains no bfw information. Therefore, the same derived stream network (DSN) that helped identify mainstems [4] was used to identify channels with bfw of 5-25 m. The workflow in ArcMap was similar to that described for mainstems in that it involved:

1. selecting (“by attributes”) DSN reaches 5-25 m in bfw;
2. exporting the selected reaches to a new shapefile with the projection defined to match the WCD;
3. selecting (“by location”) WCD reaches that intersect a 50 m buffer around the exported DSN reaches;
4. de-selecting WCD reaches for which WC_GNIS_NR = 0;
5. adding a new field to the WCD layer to identify which reaches were selected; and
6. editing this field to remove any mistakenly selected reaches (e.g., reaches <5 m or >25 m bfw that are located within the 50 m buffer, and reaches 5-25 m bfw that are located within the 50 m buffer but are not connected to mainstem reaches downstream).

As with mainstems, we used the resulting shapefile of tributaries as a basis for adding a riparian buffer extending 100 m landward from each bank. We then identified and omitted extraneous polygons (e.g., tributary areas that overlapped nearshore and estuary habitat areas).

Floodplain Habitat Area

Floodplain boundaries were based on a shapefile, fpu2, created for a multi-agency study of Puget Sound floodplains [5]. The fpu2 shapefile contains floodplain polygons for river reaches with drainage areas >50 km^2^. The polygons were derived from the high resolution National Hydrography Dataset (NHD; http://nhd.usgs.gov/data.html) and the 10 m National Elevation Dataset (NED; http://ned.usgs.gov/) by:

1. extracting river reaches in the Puget Sound region from the high resolution NHD;
2. ascertaining drainage areas for each river reach using NHDPlus, Version 2 (http://www.horizon-systems.com/nhdplus/);
3. determining river reach elevations by overlaying the NED with the reach lines;
4. creating a grid of elevation differences between each NED pixel and the closest point on a river reach;
5. defining floodplains as pixels with elevations <10 m above river reaches with drainage areas >50 km^2^; and
6. converting floodplain pixels from raster to polygon format (fpu2).

We used the “Identity” tool in ArcMap to distinguish floodplain areas that overlapped nearshore and estuarine areas, as defined above. These areas of overlap were not included in subsequent analyses.

Basin Habitat Area

The basin (i.e., whole watershed) boundaries were based on a national geodatabase called the Watershed Boundary Dataset (WBD; http://water.usgs.gov/GIS/huc.html). We downloaded the WBD for Washington State from the Washington State Department of Ecology website (http://www.ecy.wa.gov/services/gis/data/data.htm). Within this geodatabase, we used the WBD_HU10 layer, which represented the “fifth level” hydrologic unit boundaries. This statewide layer contained 494 polygons, 161 of which overlapped the Puget Sound-specific LandTrendr extent. The majority of this overlap was caused by slight misalignments between the WBD_HU10 and LandTrendr layers. Therefore, of those 161 polygons, only 64 collectively bounded the 17 major watersheds in the Puget Sound region.

References

1. Anchor QEA LLC (2009) Final Geospatial Methodology Used In the PSNERP Comprehensive Change Analysis of Puget Sound: Puget Sound Nearshore Ecosystem Restoration Project. U.S. Army Corps of Engineers, Seattle District and Washington State Department of Fish and Wildlife.

2. Simenstad CA, Ramirez M, Burke J, Logsdon M, Shipman H, et al. (2011) Historical Change of Puget Sound Shorelines: Puget Sound Nearshore Ecosystem Project Change Analysis. Puget Sound Nearshore Ecosystem Restoration Project Report No. 2011-01. Published by Washington Department of Fish and Wildlife, Olympia, Washington, and U.S. Army Corps of Engineers, Seattle, Washington. Available online (pdf).

3. Beechie TJ, Greene CM, Holsinger L, Beamer EM (2006) Incorporating parameter uncertainty into evaluation of spawning habitat limitations on Chinook salmon (Oncorhynchus tshawytscha) populations. Can J Fish Aquat Sci 63: 1242-1250.

4. Davies J, Lagueux KM, Sanderson BL, Beechie TJ (2007) Modeling stream channel characteristics from drainage-enforced DEMs in Puget Sound, Washington, USA. J Am Water Resour Assoc 43: 414-426.

5. Konrad C (In review) Geospatial assessment of ecological functions and flood-related risks on floodplains along major rivers in the Puget Sound basin. USGS Science Investigation Report.
